# Machine learning-based multiparametric MRI radiomics nomogram for predicting WHO/ISUP nuclear grading of clear cell renal cell carcinoma

**DOI:** 10.3389/fonc.2024.1467775

**Published:** 2024-11-07

**Authors:** Yunze Yang, Ziwei Zhang, Hua Zhang, Mengtong Liu, Jianjun Zhang

**Affiliations:** ^1^ Department of Radiology, Baoding First Central Hospital, Baoding, China; ^2^ Department of Postgraduate, Chengde Medical University, Chengde, China; ^3^ Department of Postgraduate, Hebei Medical University, Shijiazhuang, China

**Keywords:** clear cell renal cell carcinoma, radiomics nomogram, machine learning, magnetic resonance imaging, WHO/ISUP nuclear grading

## Abstract

**Objective:**

To explore the effectiveness of a machine learning-based multiparametric MRI radiomics nomogram for predicting the WHO/ISUP nuclear grading of clear cell renal cell carcinoma (ccRCC) before surgery.

**Methods:**

Data from 86 patients who underwent preoperative renal MRI scans (both plain and enhanced) and were confirmed to have ccRCC were retrospectively collected. Based on the 2016 WHO/ISUP grading standards, patients were divided into a low-grade group (Grade I and II) and a high-grade group (Grade III and IV), and randomly split into training and testing sets at a 7:3 ratio. Radiomics features were extracted from FS-T2WI, DWI, and CE-T1WI sequences. Optimal features were selected using the Mann-Whitney U test, Spearman correlation analysis, and the least absolute shrinkage and selection operator (LASSO). Five machine learning classifiers—logistic regression (LR), naive bayes (NB), k-nearest neighbors (KNN), adaptive boosting (AdaBoost), and multilayer perceptron (MLP)—were used to build models to predict ccRCC WHO/ISUP nuclear grading. The model with the highest area under the curve (AUC) in the testing set was chosen as the best radiomics model. Independent clinical risk factors were identified using univariate and multivariate logistic regression to create a clinical model, which was combined with radiomics score (rad-score) to develop a nomogram. The model’s effectiveness was assessed using the receiver operating characteristic (ROC) curve, its calibration was evaluated using a calibration curve, and its clinical utility was analyzed using decision curve analysis.

**Results:**

Six radiomics features were ultimately selected. The MLP classifier showed the highest diagnostic performance in the testing set (AUC=0.933). Corticomedullary enhancement level (P=0.020) and renal vein invasion (P=0.011) were identified as independent risk factors for predicting the WHO/ISUP nuclear classification and were included in the nomogram with the rad-score. The ROC curves indicated that the nomogram model had strong diagnostic performance, with AUC values of 0.964 in the training set and 0.933 in the testing set.

**Conclusion:**

The machine learning-based multiparametric MRI radiomics nomogram provides a highly predictive, non-invasive tool for preoperative prediction of WHO/ISUP nuclear grading in patients with ccRCC.

## 1 Introduction

Renal cell carcinoma (RCC) originates from the epithelial cells of the renal tubules and accounts for approximately 90% of all malignant kidney tumors and 2%-3% of all body malignancies (1, 2). Clear cell renal cell carcinoma (ccRCC), as the most common subtype of RCC (comprising 70%-80% of RCC cases), is associated with poor prognosis and high mortality rates (3, 4). Previous studies have shown that the prognosis of ccRCC patients is closely related to the tumor’s nuclear grade; generally, the higher the nuclear grade, the shorter the survival period (5). The grading system introduced by the World Health Organization/International Society of Urological Pathology (WHO/ISUP) in 2016 classifies grade I and II tumors as low-grade and grade III and IV tumors as high-grade. This system is widely adopted due to its practicality and clinical relevance (6).

Current research indicates that the choice of surgical method is related to the pathological nuclear grading of ccRCC (7). Clinically, patients with low-grade ccRCC may undergo relatively conservative treatments, such as radiofrequency ablation, nephron-sparing surgery, or active surveillance; whereas patients with high-grade ccRCC require radical surgical resection (8). Additionally, the higher the pathological grade, the higher the recurrence rate after surgery (9). Thus, identifying the nuclear differentiation of ccRCC is crucial for formulating clinical treatment strategies.

Currently, the histological typing and grading of renal cancer primarily rely on percutaneous renal biopsy. However, biopsy is an invasive procedure that may lead to various complications such as metastasis and bleeding (10, 11). MRI is widely used in preoperative examinations of renal cancer due to its non-invasiveness, high soft tissue resolution, and the ability to provide multi-sequence, multi-parameter, and multi-directional imaging (12). However, traditional radiological diagnoses often depend on the subjective experience of radiologists, affecting the accuracy of these indicators. Radiomics enables the high-throughput extraction of features from imaging data that are not visible to the human eye. Machine learning builds upon this by using algorithmic models to learn, analyze, and predict these extensive, high-dimensional radiomics features, thereby establishing models that can improve disease diagnosis, prognosis, and prediction accuracy (13–15). This study selects different machine learning methods to develop and validate radiomics models and establishes a nomogram model based on multi-parametric MRI radiomics and clinico-radiological features for the preoperative prediction of the WHO/ISUP grading of ccRCC, aiming to provide new methods for non-invasive clinical diagnosis, treatment selection, and prognosis assessment.

## 2 Materials and methods

### 2.1 General information

**Figure 1:**
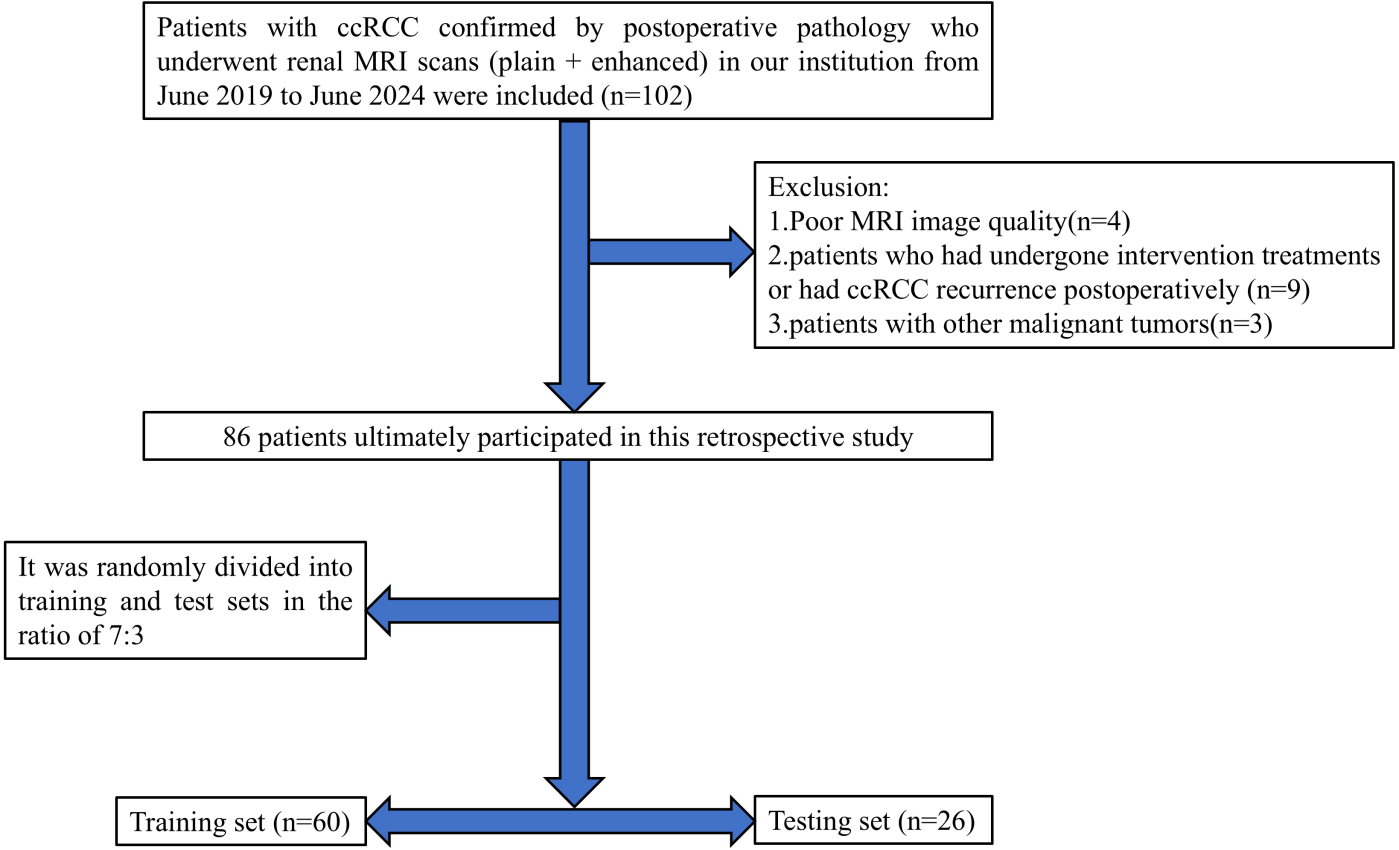
Flowchart of the study subjects based on exclusion criteria.

This retrospective study received approval from the Medical Ethics Committee of Baoding’s First Central Hospital, with patient informed consent being waived. The clinical and imaging data of patients with ccRCC confirmed by postoperative pathology who underwent renal MRI plain scan and enhanced scan in Baoding’s First Central Hospital from June 2019 to June 2024 were retrospectively collected. Inclusion criteria were: (1) patients who underwent partial or radical nephrectomy and were pathologically diagnosed with ccRCC; (2) MRI scans of the kidney performed within two weeks before surgery; (3) complete clinical, pathological, and imaging data available for the patients. Exclusion criteria included: (1) poor MRI image quality affected by artifacts from breathing, motion, etc.; (2) patients who had undergone intervention treatments or had ccRCC recurrence postoperatively; (3) patients with other malignant tumors. Ultimately, 86 patients met the inclusion criteria, consisting of 57 males and 29 females, aged between 36 and 85 years (average age: 59.29 ± 11.43). According to the 2016 WHO/ISUP grading standards, patients were classified into low-grade (grades I and II, 66 cases) and high-grade (grades III and IV, 20 cases) groups. Cases were randomly divided into training (n=60) and testing (n=26) sets in a 7:3 ratio. A flowchart of the study subjects is shown in **Figure 1**.

### 2.2 Examination methods

All MRI examinations were performed using a Philips Achieva TX 3.0 T system. Patients were positioned supine and instructed to breathe calmly. A 16-channel phased-array body coil was used to cover the kidneys and lesions thoroughly. MRI parameters were as follows: (1) axial fat-suppressed T2WI sequence, TR 1089ms, TE 80ms, interslice gap 1mm, slice thickness 5mm, matrix 332×332, FOV 350mm×400mm; (2) axial DWI, TR 1145ms, TE 62ms, b-value 1000s/mm^2^; slice thickness, interslice gap, and FOV as above; (3) DCE-MRI, scanning parameters: TR 5.8ms, TE 1.7ms, slice thickness 5mm, interslice gap 0.78 mm, FOV 220mm × 189mm. The contrast agent used for the enhanced scans was gadopentetate dimeglumine (Gd-DTPA, Bayer Healthcare Pharmaceuticals, Germany) at a dose of 0.1mmol/kg, administered intravenously at 2ml/s using a high-pressure injector, immediately followed by a 20 ml saline flush at the same rate.

### 2.3 Clinical and radiological feature analysis

Clinical data such as age, gender, tumor location (left, right), maximum tumor diameter, presence of hematuria, smoking history, hypertension history, and diabetes history were obtained from medical records.

MRI radiological feature analysis was conducted by two radiologists specializing in abdominal imaging in a double-blind manner, including Physician A with 3 years of experience and Physician B with 20 years of experience. Any discrepancies were resolved through consultation. Evaluation criteria included: boundaries (clear, unclear), pseudocapsule (present, absent), cystic necrosis (present, absent), renal vein invasion (present, absent), involvement of the renal sinus (present, absent), distant metastasis (present, absent), intratumoral vessels (present, absent), lesion DWI signal intensity (compared to renal parenchyma: isointense, hyperintense), and corticomedullary enhancement level (solid part of the tumor having lower, higher, or similar enhancement compared to renal parenchyma). The ADC values of the tumor parenchyma were manually measured three times for each lesion to minimize error.

### 2.4 Tumor segmentation and feature extraction

All MRI images were exported from the Picture Archiving and Communication System (PACS) in DICOM format. Physician A used 3D Slicer software version 5.4.0 (www.slicer.org) to manually delineate the lesions layer by layer along the edges, drawing regions of interest (ROI) as shown in **Figure 1**, which included axial fat-suppressed T2-weighted imaging (FS-T2WI), diffusion-weighted imaging (DWI), and corticomedullary phase contrast-enhanced T1-weighted imaging (CE-T1WI). Before feature extraction, grayscale values were normalized. Subsequently, a total of 2553 radiomics features were automatically extracted using the Pyradiomics module within the 3D Slicer software.

### 2.5 Feature selection and machine learning

All radiomics features underwent Mann-Whitney U testing for feature selection, retaining only those features with a p-value <0.05. For features with high redundancy, Spearman correlation analysis was used to assess inter-feature correlations, retaining one feature from any pair with a correlation coefficient greater than 0.9. The optimal subset of features was then identified using least absolute shrinkage and selection operator (LASSO) regression. These selected features were incorporated into five machine learning classifiers: logistic regression (LR), naive bayes (NB), k-nearest neighbors (KNN), adaptive boosting (AdaBoost), and multilayer perceptron (MLP). The classifier with the highest area under curve (AUC) in the test set was selected for radiomics modeling, which was then converted into a corresponding radiomics score (Rad-score).

### 2.6 Construction of the nomogram model

In the training set, both univariate and multivariate logistic regression analyses were performed on the clinical and radiological features to identify statistically significant features (P < 0.05) for constructing the clinical model. Finally, a nomogram model was built based on the clinically independent risk factors and the rad-score.

### 2.7 Statistical analysis

**Figure 2:**
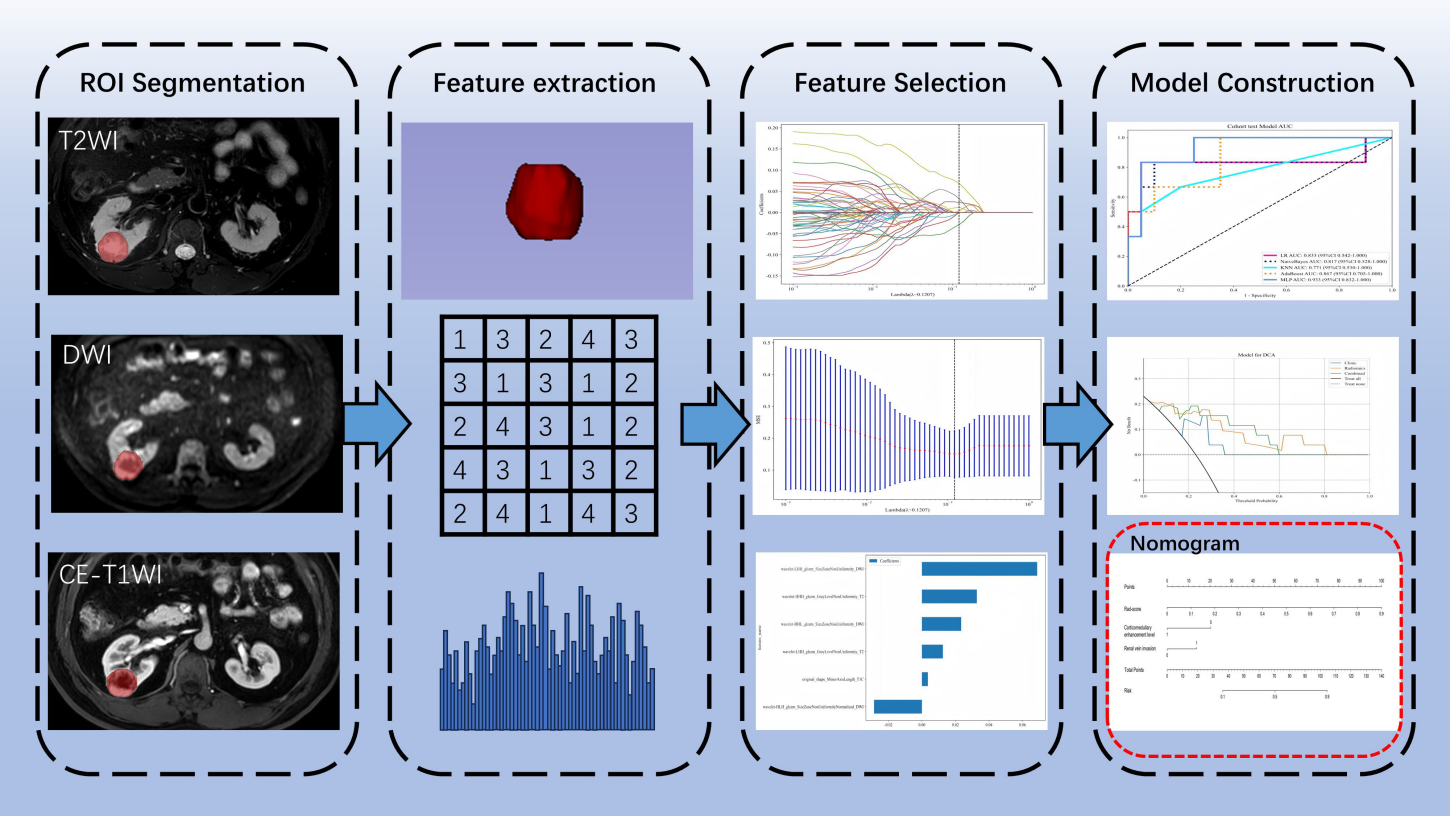
Workflow of radiomics analysis of this study.

Statistical analyses were conducted using SPSS version 26.0 and R version 4.3.2, with a p-value <0.05 considered statistically significant. Continuous variables were analyzed using independent samples t-tests or Mann-Whitney U tests. Categorical variables were analyzed using the chi-square test. 5-fold cross validation was used in model training. The effectiveness of the models was assessed by calculating the AUC of the receiver operating characteristic (ROC) curve. Differences between models were compared using the DeLong test. Model calibration performance was evaluated using calibration curves. The clinical utility of the models was assessed using decision curve analysis (DCA). **Figure 2** shows the workflow of radiomics analysis of this study.

## 3 Results

### 3.1 General data comparison

This study included a total of 86 ccRCC patients, comprising 66 low-grade (WHO grades I and II) and 22 high-grade (WHO grades III and IV) patients. Significant statistical differences were observed in the training and testing groups regarding maximum tumor diameter, tumor boundaries, renal sinus involvement, and venous thrombus (P < 0.05). Refer to **Table 1**.

**Table 1:** Comparison of general patient data (S).

Parameter	Training Set			Test Set		
	Low Grade	High Grade	P-value	Low Grade	High Grade	P-value
Age (years)	57.60 ± 11.88	65.38 ± 8.64	0.032	59.25 ± 12.64	59.50 ± 3.78	0.963
Gender			0.784			0.971
Female	15 (31.91)	3 (23.08)		9 (45.00)	2 (33.33)	
Male	32 (68.09)	10 (76.92)		11 (55.00)	4 (66.67)	
Maximum Tumor Diameter (cm)	3.57 ± 2.20	7.06 ± 2.78	<0.001	3.76 ± 1.33	7.70 ± 2.55	<0.001
Tumor location			0.682			
Left	20 (42.55)	7 (53.85)		9 (45.00)	1 (16.67)	0.440
Right	27 (57.45)	6 (46.15)		11 (55.00)	5 (83.33)	
Boundaries			<0.001			
Clear	38 (80.85)	2 (15.38)		17 (85.00)	1 (16.67)	0.007
Unclear	9 (19.15)	11 (84.62)		3 (15.00)	5 (83.33)	
Smoking history	16 (34.04)	5 (38.46)	1.000	4 (20.00)	1 (16.67)	
History of hypertension	23 (48.94)	8 (61.54)	0.623	5 (25.00)	2 (33.33)	1.000
History of diabetes	10 (21.28)	4 (30.77)	0.73	5 (25.00)	1 (16.67)	1.000
Hematuria	4 (8.51)	2 (15.38)	0.835	3 (15.00)	1 (16.67)	1.000
Pseudocapsule	18 (38.30)	8 (61.54)	0.238	8 (40.00)	5 (83.33)	1.000
Renal sinus involvement	8 (17.02)	10 (76.92)	<0.001	4 (20.00)	5(83.33)	0.163
DWI signal intensity			0.036			0.018
isosignal	16 (34.04)	0		5 (25.00)	0	0.440
high signal	31 (65.96)	13 (100.00)		15 (75.00)	6 (100.00)	
Intratumoral vessels	12 (25.53)	9 (69.23)	0.009	6 (30.00)	4 (66.67)	
ADC value (mm^2^/s)	1.87 ± 0.41	1.48 ± 0.63	0.011	1.73 ± 0.52	1.45 ± 0.62	0.254
Renal vein invasion	1 (2.13)	7 (53.85)	<0.001	0	4 (66.67)	0.279
Cystic necrosis	32 (68.09)	11 (84.62)	0.411	15 (75.00)	5 (83.33)	<0.001
Corticomedullary Enhancement Level			0.021			1.000
Lower than renal parenchyma	17 (36.17)	10 (76.92)		6 (30.00)	2 (33.33)	1.000
Higher than or equal to renal parenchyma	30 (63.83)	3 (23.08)		14 (70.00)	4 (66.67)	

### 3.2 Performance evaluation of machine learning classifiers

**Figure 3:**
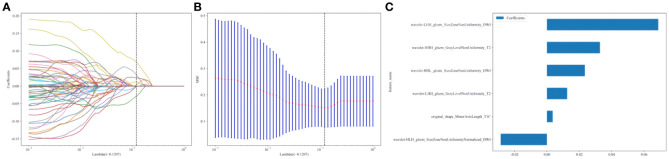
**(A–C)** Radiomics features selection and establishment of rad-score based on LASSO algorithm.10-fold cross-validation coefficients and MSE **(A, B)**. Rad-score histogram based on the selected features **(C)**.

**Figure 4:**
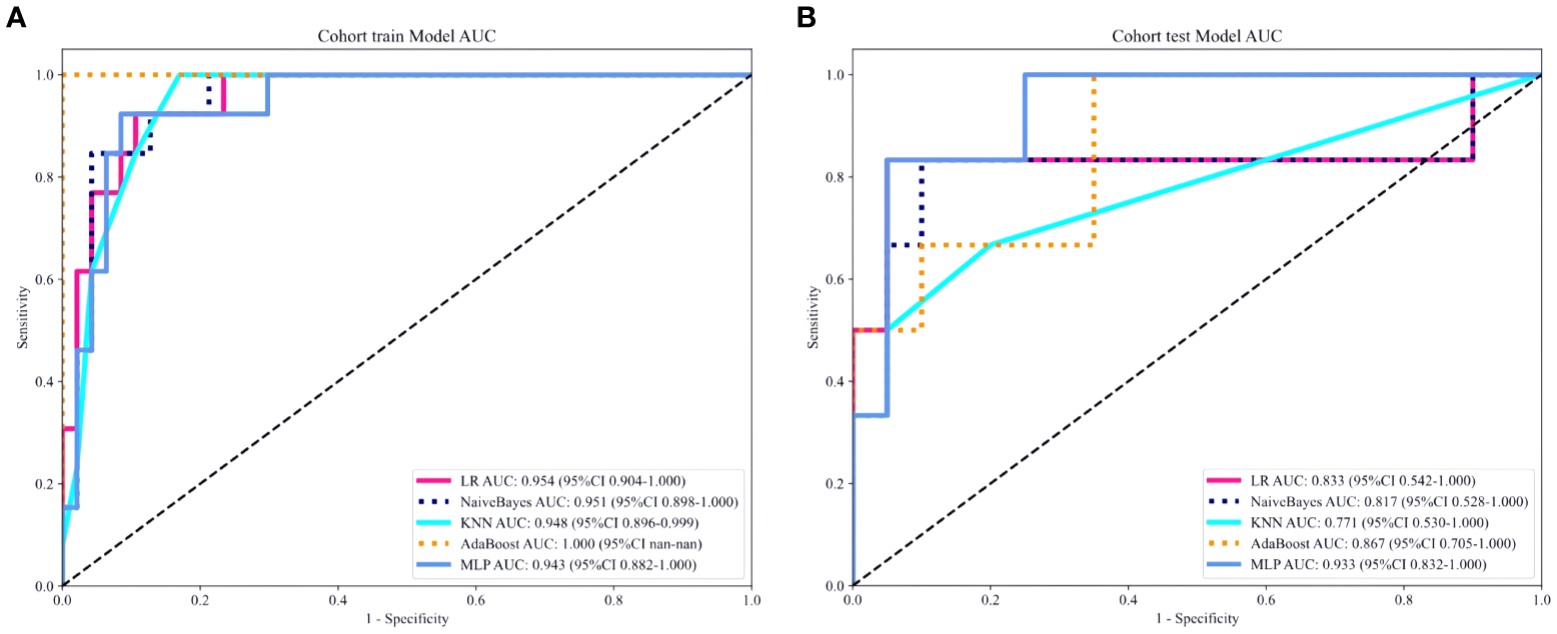
**(A, B)** ROC curves of five different machine learning algorithms predicting the grading of ccRCC in the training set **(A)** and the testing set **(B)**.

A total of 2553 radiomics features were extracted from the axial FS-T2WI, DWI, and CE-T1WI images. After performing Mann-Whitney U testing, 1189 features with a P < 0.05 were retained. Following Spearman correlation analysis, 316 features remained. Ultimately, 6 features were selected through LASSO regression. Based on these features, five machine learning classifiers were constructed and further analyzed (**Figures 3A–C**). ROC curve analysis showed that the AUC values for the five machine learning classifiers (LR, NB, KNN, AdaBoost, MLP) ranged from 0.943 to 1.000 in the training set, and from 0.771 to 0.933 in the testing set (see **Figures 4A, B**). In the training set, the best classifier was AdaBoost, with an AUC of 1.000; however, in the testing set, the best classifier was the MLP. The MLP’s performance metrics, including AUC, sensitivity, specificity, and accuracy were 0.933, 0.667, 0.950, and 0.800, respectively (see **Table 2**). Although AdaBoost performed well in the training set, it showed signs of overfitting in the testing set. To ensure model stability and consistency, MLP was chosen as the best radiomics model.

**Table 2:** Comparison of diagnostic performance of five machine learning classifiers in training and test sets.

Machine Learning Classifier	Training Set				Test Set			
	AUC(95%CI)	Sensitivity	Specificity	Accuracy	AUC(95%CI)	Sensitivity	Specificity	Accuracy
LR	0.954(0.904-1.000)	0.846	0.894	0.883	0.833(0.542-1.000)	0.667	0.950	0.885
KNN	0.948(0.896-0.999)	0.846	0.894	0.883	0.771(0.530-1.000)	0.500	0.950	0.846
MLP	0.943(0.882-1.000)	0.846	0.915	0.900	0.933(0.832-1.000)	0.667	0.950	0.885
NaiveBayes	0.951(0.898-1.000)	0.769	0.957	0.917	0.817(0.528-1.000)	0.667	0.900	0.846
AdaBoost	1.000(1.000-1.000)	0.923	1.000	0.983	0.867(0.705-1.000)	0.833	0.650	0.692

### 3.3 Model construction and validation

**Figure 5:**
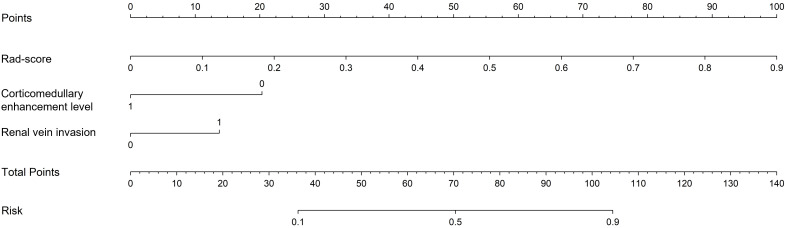
Nomogram used to predict ccRCC grading: includes radiomics score, corticomedullary enhancement level and renal vein invasion.

After univariate and multivariate logistic regression analyses of traditional clinical and radiological indicators, two independent risk factors were identified for the construction of the clinical model: corticomedullary enhancement level and renal vein invasion (see **Table 3**). A nomogram model was constructed by combining the clinical model and the Rad-score from the best radiomics model (see **Figure 5**). Comparisons of the AUC values, specificity, sensitivity, and accuracy among the three models showed that the nomogram model outperformed the other models in both the training group (AUC: 0.964) and the testing group (AUC: 0.933), as detailed in **Table 4**.

**Table 3:** Univariate and multivariate logistic regression analysis of clinical and radiological features.

Parameter	Univariate Logistic Regression Analysis		Multivariate Logistic Regression Analysis	
	OR (95%CI)	P-value	OR (95%CI)	P-value
Age	1.010 (1.002-1.017)	0.032	1.005 (0.999-1.011)	0.148
Maximum Tumor Diameter	1.084 (1.054-1.115)	0.000	0.999 (0.958-1.042)	0.976
ADC Value	0.757 (0.634-0.902)	0.010	0.909 (0.776-1.064)	0.312
DWI Signal Intensity	1.344 (1.107-1.631)	0.014	1.195 (1.015-1.408)	0.074
Corticomedullary Enhancement Level	0.756 (0.637-0.898)	0.008	0.810 (0.699-0.938)	0.020
Intratumoral Vessels	1.385 (1.162-1.652)	0.003	0.978 (0.818-1.169)	0.833
Renal Sinus Involvement	1.623 (1.374-1.916)	0.000	1.043 (0.836-1.302)	0.750
Boundaries	1.649 (1.409-1.929)	0.000	1.194 (0.970-1.468)	0.158
Renal vein invasion	2.137 (1.737-2.630)	0.000	1.523 (1.168-1.988)	0.011

**Table 4:** Comparison of three models.

Model	Training Set				Test Set			
	AUC(95%CI)	Sensitivity	Specificity	Accuracy	AUC(95%CI)	Sensitivity	Specificity	Accuracy
Clinical Model	0.867(0.757-0.977)	0.538	0.979	0.883	0.866(0.656-1.000)	0.167	1.000	0.808
Radiomics Model	0.943(0.882-1.000)	0.846	0.915	0.900	0.933(0.832-1.000)	0.667	0.950	0.885
Nomogram Model	0.964(0.923-1.000)	0.923	0.872	0.883	0.942(0.822-1.000)	0.667	1.000	0.923

**Figure 6:**
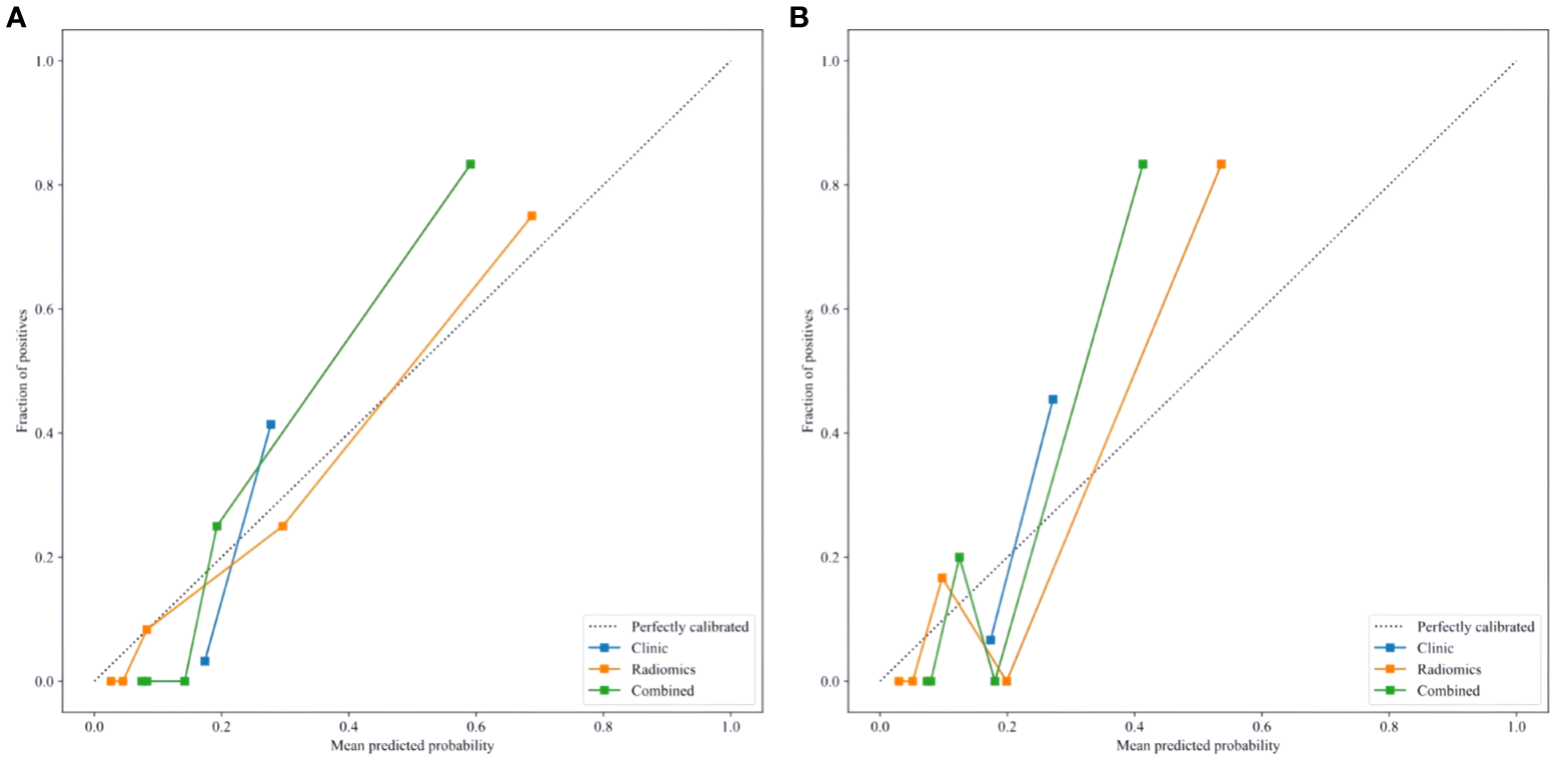
**(A, B)** Calibration curves of the nomogram in the training **(A)** and testing set **(B)**.

**Figure 7:**
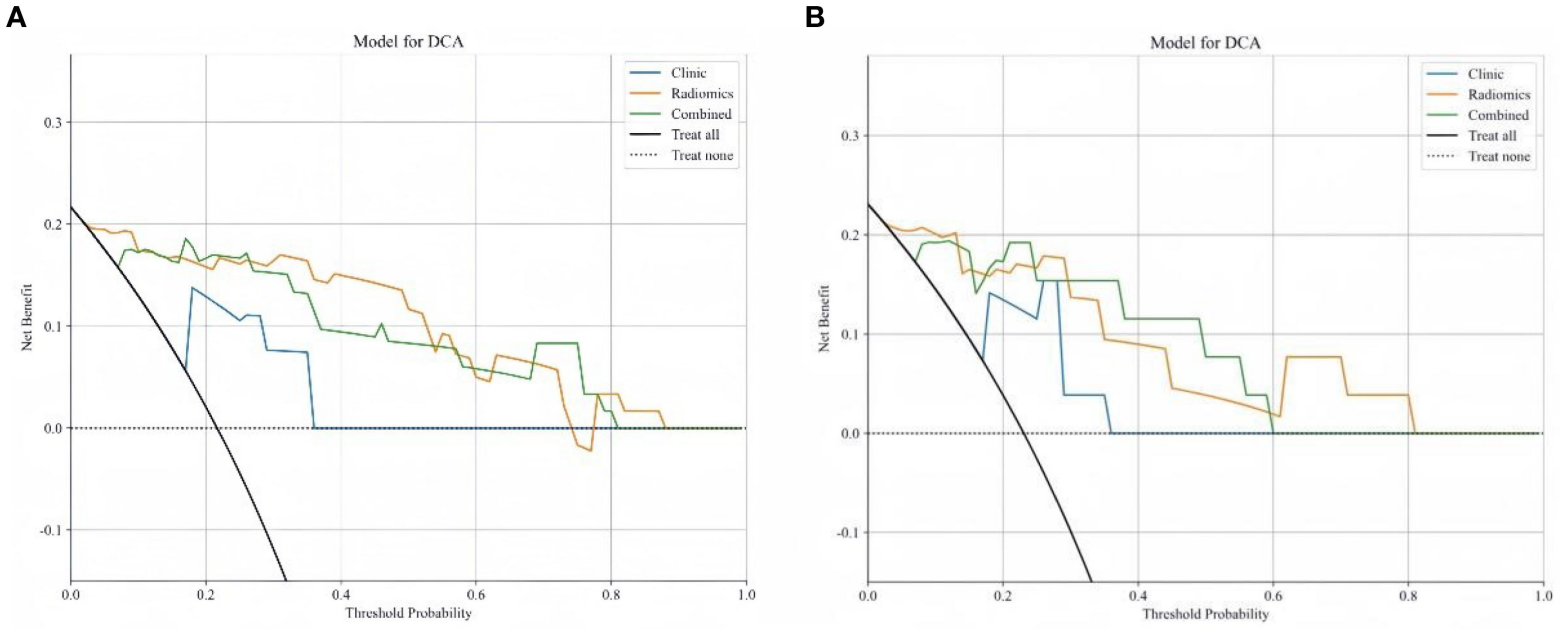
**(A, B)** DCA curves for training set **(A)** and testing set **(B)**.

Delong test results indicated that the AUC values of the three models in both the training and testing set were statistically significant (P < 0.05). **Figures 6A, B** show the calibration curves in the training and testing set. DCA results demonstrated that the nomogram model provided a higher net benefit within certain thresholds (**Figures 7A, B**), suggesting that the nomogram model has higher clinical utility in the preoperative prediction of ccRCC WHO/ISUP grading.

## 4 Discussion

As the most common subtype of RCC, ccRCC is known for its aggressiveness and high malignancy grade (16). Previous studies have demonstrated that nuclear grading of tumor cells is the most effective independent prognostic factor for survival in ccRCC patients; higher nuclear grades are associated with poorer prognoses (17, 18). Additionally, the nuclear grade of ccRCC correlates with the tumor’s biological behavior and metastatic potential. Patients with high-grade ccRCC are prone to systemic bone metastases and renal vein invasion, and the rate of local tumor recurrence is also higher, severely affecting patient outcomes (19, 20). Therefore, preoperative prediction of nuclear grading in ccRCC patients is crucial for clinical decision-making.

Previous studies have identified statistically significant differences in radiological features such as tumor morphology, borders, intensity of cortical-medullary enhancement, and distant metastasis between high and low-grade ccRCC groups (21–23). In the research conducted by Li et al. (21), shape, margins, and necrosis were identified as independent predictors of high-grade ccRCC. Lesions in low-grade ccRCC typically exhibit regular margins and are generally round, while high-grade ccRCC lesions often display aggressive characteristics, with irregular shapes, indistinct borders, accompanied by necrosis, perirenal fat infiltration, or distant metastasis. Wei et al. (22) found that low-grade ccRCC features relatively mature arteries, rapid blood flow, and enhanced vascular permeability, whereas high-grade ccRCC is characterized by pronounced malignancy, accelerated tumor growth, and uneven distribution of blood supply, findings that align with those reported by Coy et al. (23).

However, univariate and multivariate logistic regression analyses in this study indicate that only renal vein invasion and corticomedullary enhancement level are independent risk factors for predicting the nuclear grading of ccRCC. This discrepancy may be due to the small sample size of this study and potential selection bias in the data. High-grade ccRCC tumors often show less corticomedullary phase enhancement compared to normal renal parenchyma, consistent with findings by Halefoglu et al. (24). This may be due to the rapid growth rate of high-grade ccRCC tumors, which likely suffer from relative ischemia, leading to more frequent micro-necrosis that affects enhancement patterns. Furthermore, high-grade ccRCC is more likely to cause renal vein invasion because these tumors are highly differentiated, aggressive, and thus more prone to venous and lymphatic metastasis, leading to poorer prognoses (25).

In recent years, studies have confirmed the significant value of radiomics in the pathological grading of ccRCC (26–31). Sun et al. (30) extracted radiomics features from enhanced CT images of ccRCC patients and constructed a Support Vector Machine (SVM) model, which achieved good results in distinguishing between low-grade and high-grade ccRCC, with an AUC of 0.910. Yet, they only used a single machine learning algorithm and did not compare the predictive efficacy between different machine learning classifiers. Gao et al. (31) analyzed radiological signs in ccRCC patients and extracted radiomics features from CT images to calculate rad-score and build a nomogram model. Their results indicated that the nomogram model had the best predictive performance in the pathological grading of ccRCC, with an AUC of 0.941. While most previous studies have relied on a single radiomics model for prediction, this study integrates multiple machine learning algorithms to construct a more comprehensive predictive model, significantly enhancing its accuracy. Moreover, compared with most previous CT studies, MRI offers superior soft tissue contrast and enables multi-parametric imaging, allowing for a more thorough assessment of the radiological characteristics of ccRCC.

This study employed five machine learning classifiers (LR, KNN, MLP, NB, and AdaBoost) to develop and validate a machine learning-based multi-parametric MRI radiomics nomogram model for the preoperative non-invasive and personalized prediction of ccRCC patients’ WHO/ISUP nuclear grading. The results indicated that MLP had the best predictive performance in the testing set (AUC=0.933, 95%CI=0.832-1.000), hence it was selected as the optimal radiomics model. As a machine learning algorithm inspired by the human brain, the Multi-Layer Perceptron (MLP) learns patterns and builds models from large datasets via deep learning, optimizing parameters during the training process. By leveraging the interplay between parameters and activation functions, MLP excels in feature extraction, demonstrating strong learning capabilities and robustness (32). Furthermore, this study aimed to construct a visual clinical prediction model by integrating rad-score with corticomedullary enhancement level and renal vein invasion to establish a nomogram model. The results confirmed that this nomogram model exhibited substantial potential in both the training set (AUC: 0.964) and the testing set (AUC: 0.942), surpassing both the single clinical model (AUC values of 0.867 and 0.866 respectively) and the radiomics model (AUC values of 0.943 and 0.933 respectively), highlighting its potential in predicting WHO/ISUP nuclear grading of ccRCC patients.

Limitations of this study include:

1. This is a single-center retrospective study with a small sample size, inevitably introducing bias in data selection. Future studies will require multi-center, large-sample, prospective research to validate the model’s stability and enhance clinical predictive capability.
2. Current practices in radiomics, such as image acquisition, tumor segmentation, and feature extraction, lack consistency, and manual ROI segmentation introduces certain errors. Establishing unified standards will be essential to further improve accuracy.
3. Most cases in this study had lesion diameters greater than 4 cm, while research on small renal cancers (≤4 cm) is particularly crucial for practical clinical work, necessitating further studies to expand the sample size and validate its value.

In conclusion, the machine learning-based multi-parametric MRI radiomics nomogram has demonstrated significant predictive value in the preoperative non-invasive prediction of WHO/ISUP nuclear grading in ccRCC patients. It holds promise for providing strategies for non-invasive diagnosis and personalized treatment of ccRCC patients, potentially improving long-term outcomes.

## Data Availability

The raw data supporting the conclusions of this article will be made available by the authors, without undue reservation.
